# Does One Size Fit All? External Validation of the rCAST Score to Predict the Hospital Outcomes of Post-Cardiac Arrest Patients Receiving Targeted Temperature Management

**DOI:** 10.3390/jcm12010242

**Published:** 2022-12-28

**Authors:** Chao-Hsien Chen, Chieh-Jen Wang, I-Ting Wang, Sheng-Hsiung Yang, Ya-Hui Wang, Chang-Yi Lin

**Affiliations:** 1 Division of Pulmonary and Critical Care Medicine, Department of Internal Medicine, MacKay Memorial Hospital, Taipei 104217, Taiwan; 2 Department of Medicine, MacKay Medical College, New Taipei City 25245, Taiwan; 3 Medical Research Center, Cardinal Tien Hospital, New Taipei City 23148, Taiwan

**Keywords:** mortality, neurologic outcome, out-of-hospital cardiac arrest, post-cardiac arrest syndrome, rCAST, targeted temperature management

## Abstract

The revised post-Cardiac Arrest Syndrome for Therapeutic hypothermia (rCAST) score was proposed to predict neurologic outcomes and mortality among out-of-hospital cardiac arrest (OHCA) patients. However, it has rarely been validated outside Japan. Therefore, this study aimed to investigate this issue. All adult patients admitted to our medical intensive care unit for targeted temperature management (TTM) between July 2015 and July 2021 were enrolled. Their medical records were retrieved, and rCAST scores were calculated. A total of 108 post-cardiac arrest syndrome (PCAS) patients who received TTM were analyzed. According to the rCAST score, 49.1%, 50.0%, and 0.9% of the patients were classified as low, moderate, and high severity, respectively. The areas under the curves for the rCAST score were 0.806 (95% confidence interval [CI]: 0.719–0.876) and 0.794 (95% CI: 0.706–0.866) to predict poor neurologic outcomes and mortality at day 28, respectively. In contrast to the original report, only low-severity patients had favorable neurologic outcomes. The rCAST score showed moderate accuracy in our OHCA patients with PCAS who received TTM to predict poor neurologic outcomes and mortality at day 28.

## 1. Introduction

Treatment for sudden cardiac arrest remains challenging. The reported global annual incidence of emergency medical services (EMS)-treated out-of-hospital cardiac arrest (OHCA) ranges from 30.0 to 97.1/100,000 population [1] of whom only 22.0% survive to hospital admission and 8.8% survive to hospital discharge. Moreover, approximately 22% of OHCA survivors in the United States have poor neurological outcomes [2] and OHCA remains a leading cause of disability-adjusted life years [3,4].

Targeted temperature management (TTM) has been included as part of the standard management for post-cardiac arrest syndrome (PCAS) since the cornerstone reports from Bernard et al. and the HACA study group [5,6]. Targeted hypothermia (TTM between 32~34 °C) can effectively prevent further hypoxic-ischemic brain injury and improve survival and neurologic outcomes [7]. However, the complex clinical scenario, use of sedation, and altered metabolic status during TTM [8,9] make the early prediction of neurological outcomes in PCAS patients difficult [10,11] especially for those who wish to withdraw life-sustaining treatment if a grave outcome is inevitable [12]. The revised post-Cardiac Arrest Syndrome for Therapeutic hypothermia (rCAST) score proposed by Nishikimi et al. [13,14] is a clinical score that can easily be calculated after the return of spontaneous circulation (ROSC) in OHCA patients. It has been demonstrated to be a good predictor of neurologic outcomes and mortality in OHCA patients receiving TTM in Japan [14].

The optimal temperature zone control for PCAS patients remains controversial. Nielsen et al. reported that targeted normothermia (TTM between 36~37 °C) was effective in managing PCAS patients [15]. However, some studies have reported a slight increase in in-hospital mortality and fever episodes with the use of targeted normothermia [16,17]. In a retrospective cohort study, Callaway et al. found that targeted hypothermia was more beneficial for patients with severe post-cardiac arrest illness [18]. Nishikimi et al. also found that targeted hypothermia was more effective in improving neurologic outcomes than targeted normothermia in patients with moderate severity according to the rCAST score [19]. Using the rCAST score to assess who should receive TTM is appealing; however, its accuracy has not been validated in settings other than in Japan. Our institute has integrated TTM into PCAS patient management since 2007, initially for OHCA patients and then for in-hospital cardiac arrest (IHCA) patients [20,21]. This study aimed to validate the accuracy of the rCAST score for OHCA patients at our institute.

## 2. Materials and Methods

### 2.1. Study Design and Patient Selection

This retrospective observational study was conducted at MacKay Memorial Hospital (MMH). MMH is a tertiary hospital with two locations in Taipei, with a total of 1621 beds and 119 intensive care unit (ICU) beds. All patients admitted to medical ICUs for TTM between 1 July 2015, and 31 July 2021, were enrolled for screening. The inclusion criteria included: (1) PCAS patients after OHCA, (2) Older than 18 years of age, and (3) Receiving TTM. The patients (1) after IHCA, (2) due to traumatic cardiac arrest, (3) not receiving TTM, and (4) with any missing data needed to calculate the rCAST score were excluded. Electronic medical records were reviewed, and baseline patient profiles, co-morbidities, laboratory data, Acute Physiology and Chronic Health Evaluation (APACHE) II score, treatment received, and hospital outcomes were collected for analysis. The witness or not, the initial rhythm of cardiac arrest, and resuscitation duration before arrival hospital were coded according to the EMS records. The period of resuscitation effort was defined from starting chest compression to sustained ROSC (not requiring chest compressions for 20 consecutive minutes) [22]. The intra-aortic balloon pump (IABP) was indicated if a physician-in-charge judged cardiovascular disease-related cardiogenic shock. The rCAST score was calculated with the following five parameters before initiating TTM: initial rhythm of cardiac arrest, duration from arrest to ROSC, pH of arterial blood gas, lactate, and motor Glasgow Coma Scale (GCS) score [14]. The variables for rCAST were obtained during resuscitation or within 15 min after ROSC. The 1 mg/dL lactate is equivalent to 0.111 mmol/L. The patients were classified into the following categories according to rCAST score: low severity (rCAST ≤ 5.5), moderate severity (rCAST, 6–14), and high severity (rCAST ≥ 14.5) [14]. This study was approved by the Institutional Review Boards of MMH (approval no. 21MMHIS012e).

### 2.2. Therapeutic Hypothermia Protocol at MMH

At MMH, TTM is considered for every comatose (GCS score ≤ 8) PCAS patient admitted to the ICU, with either OHCA or IHCA, if not contraindicated. Contraindications include pregnancy, initial body temperature < 30 °C, terminal illness, intracranial bleeding, traumatic cardiac arrest, or having do-not-resuscitate orders. TTM is started initially with 4 °C Ringer’s lactate solution 30 mL/kg for induction, followed by a surface cooling device (Arctic Sun Model 2000/5000, Medivance, Louisville, CO) targeted at 33 °C for 24–48 h, using esophageal temperature measurement. Rewarming is set at a rate of 0.15 °C per hour with a target body temperature of 36.5 °C. Additional temperature control to avoid fever is allowed. Magnesium sulfate infusion with 10 g/day for one day is routinely given to prevent patients from shivering [23]. Propofol, fentanyl, succinylcholine, and atracurium can be used if required by the physician in charge.

### 2.3. Outcome Measurements

The primary outcome was the neurologic function at day 28 or discharge. A good neurologic outcome was defined as a Glasgow–Pittsburgh cerebral performance category (GP-CPC) of 1–2 (good cerebral performance or moderate cerebral disability), and a poor neurologic outcome was defined as a GP-CPC of 3–5 (severe cerebral disability, coma, or death) [24,25]. (Table S1) The secondary outcome was mortality on day 28.

### 2.4. Statistical Analysis

We validated the rCAST score to predict outcomes in OHCA patients. The predictive accuracy of the rCAST score was calculated using the area under the receiver operating characteristic curve (AUC). High accuracy was defined as an AUC > 0.9, moderate accuracy as an AUC 0.7–0.9, and low accuracy as an AUC < 0.7 [26]. Categorical variables were presented as numbers (percentage) and compared using the chi-squared or Fisher’s exact test, as appropriate. The normality of continuous variables was assessed using Shapiro–Wilk’s test. Normally distributed continuous variables were presented as mean ± standard deviation and non-normally distributed variables as median (interquartile range). The independent samples t-test was used to compare two normally distributed continuous variables, and the Mann-Whitney U test was used for non-normally distributed variables. All p values were two-sided, and *p* < 0.05 indicated a statistically significant difference. The statistical analyses were performed using MedCalc 20.106 (MedCalc Software Ltd., Ostend, Belgium).

## 3. Results

**Figure 1:**
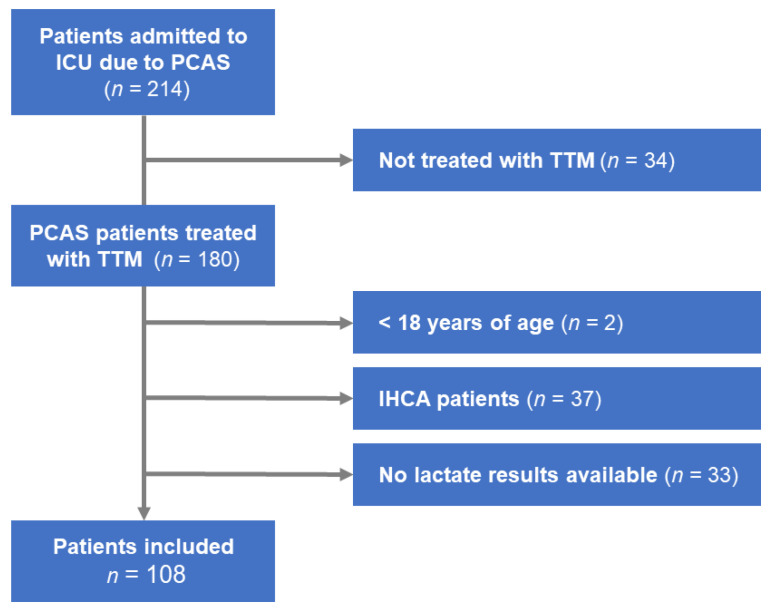
Patient enrollment flow diagram. ICU: intensive care unit, IHCA: in-hospital cardiac arrest, PCAS: post-cardiac arrest syndrome, TTM: targeted temperature management.

A total of 214 PCAS patients were admitted to an ICU during the study period, of whom 35 did not receive TTM were excluded. Another 72 patients aged < 18 years, suffered from IHCA, and with missing data needed to calculate the rCAST score were excluded. The remaining 108 OHCA patients with PCAS received TTM and were enrolled for analysis (Figure 1). 

The median age of the patients was 66.0 (55.5–77.5) years, and 66 (61.1%) of them were males. Compared to those with poor neurologic outcomes, the patients with good neurological outcomes were younger, had more bystander defibrillation, had more initial shockable rhythm, had less duration of resuscitation, had less motor of GCS < 2, had a lower rCAST score, had a lower APACHE II score, and received more IABP (Table 1). The surviving patients at day 28 were more bystander defibrillation, had more initial shockable rhythm, had less duration of resuscitation, had less motor of GCS < 2, had higher serum pH values, had lower rCAST score, had lower APACHE II score, had less diabetes, and received more percutaneous coronary intervention (PCI).

**Figure 2:**
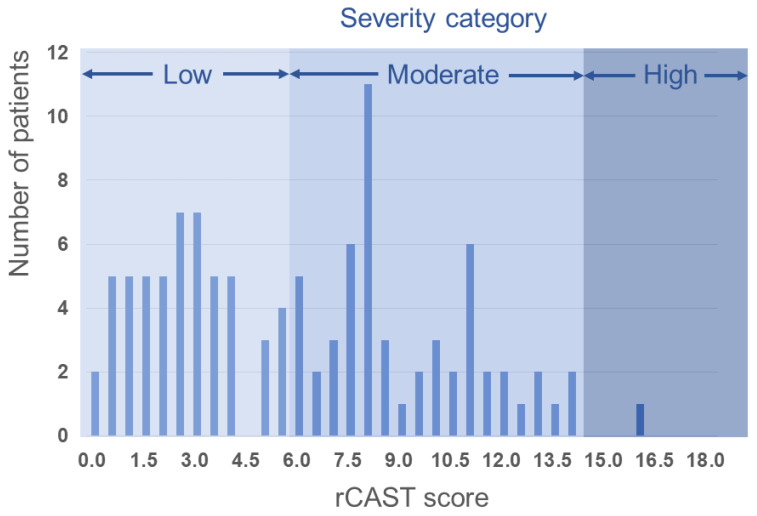
The distribution of the rCAST score and the three severity categories.

**Figure 3:**
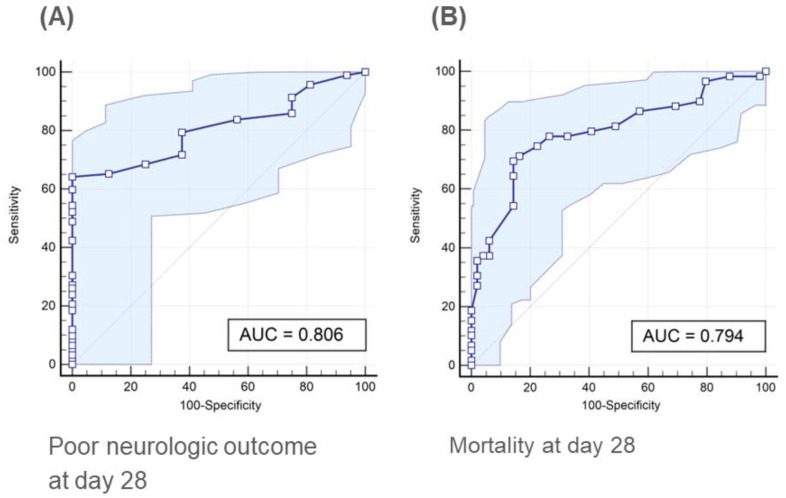
Receiver operating characteristic curves of rCAST score for predicting poor neurologic outcome (**A**) or mortality (**B**) at day 28. Light blue areas represent the 95% confidence interval. AUC: area under the curve.

**Figure 4:**
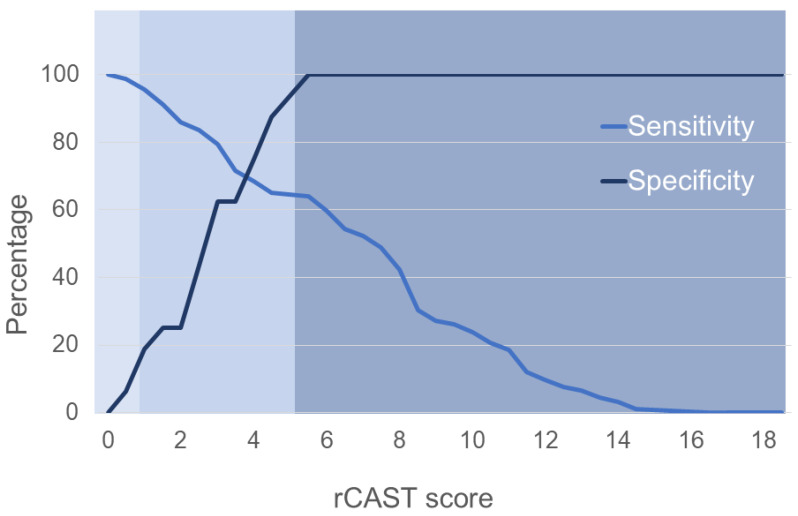
Sensitivity and specificity of using the rCAST score to predict poor neurological outcomes at day 28 in out-of-hospital cardiac arrest patients. There was decreased sensitivity and increased specificity to using a higher rCAST score as a cut point to predict the poor neurologic outcomes.

The median rCAST score was 6.0 (range, 0.0 to 16.0) (Figure 2). Fifty-three (49.1%), fifty-four (50.0%), and one (0.9%) patients were classified as low, moderate, and high severity according to the rCAST score, respectively. The AUCs for the rCAST score were 0.806 (95% confidence interval [CI]: 0.719–0.876) to predict poor neurologic outcomes (CPC 3–5) at day 28 (Figure 3A) and 0.794 (95% CI: 0.706–0.866) to predict mortality at day 28 (Figure 3B). rCAST scores showed moderate accuracy in predicting either a poor neurologic outcome or mortality at day 28. The rCAST score with 95% sensitivity and specificity to predict a poor neurological outcome at day 28 were 1.5 and 5.5, respectively (Figure 4, Table S2).

The predicted probabilities of poor neurologic outcomes on day 28 were 69.8%, 100.0%, and 100.0% with low, moderate, and high severity according to the rCAST score, and the predicted probabilities of mortality at day 28 were 28.3%, 79.6%, and 100.0%, respectively (Table 2). 

## 4. Discussion

The major findings of this study are as follows. First, most patients were classified as low and moderate severity according to the rCAST score. Second, the rCAST score had moderate predictive accuracy for a poor neurologic outcome or mortality on day 28 in the OHCA patients receiving TTM. Third, the predictive probabilities of a poor neurologic outcome in the moderate and high severity categories were 100%. 

In Nishikimi et al.’s study [14] 20.9%, 41.7%, and 37.4% of the patients were classified into low, moderate, and high severity categories, respectively. In contrast, most of our patients were classified into low and moderate severity categories (50.4% and 48.9%). Although the distribution of rCAST scores was quite different in our patients, the AUCs for rCAST (0.806 for the poor neurologic outcome and 0.794 for mortality) were compatible with the original study by Nishikimi et al. (0.892 for the 30-day poor neurologic outcome and 0.832 for mortality) [14]. A possible explanation for this may be due to differences in the characteristics between the patient groups, medical accessibility, and emergency/critical medical practice patterns. In our study, there were more witnessed OHCAs (96.0% vs. 77.2%), fewer patients with a shockable rhythm (29.0% vs. 43.7%), longer duration of resuscitation (28.0 vs. 23.0 min), higher pH (7.36 vs. 7.08), lower lactate (56.5 vs. 86.3 mg/dL), and all of our patients were treated with TTM at 33°C (100% vs. 74.6%) compared with the study of Nishikimi et al. [14]. The higher amount of witnessed OHCAs, higher pH, and lower lactate may explain why more patients had a lower rCAST score in our study. The EMS response is generally longer in rural areas than in urban or suburban areas [27]. Gräsner et al. [28] also reported that differences in EMS systems in Europe might account for at least some of the differences in OHCA incidence and survival rates. Tjelmeland et al. [29] reported that a median EMS response time of <10 min in urban areas was only achieved in 32% of European countries, and the response time was much longer in rural areas. Most of our patients live in urban or suburban areas around Taipei, whereas Nishikimi et al. included patients from urban, suburban, and rural areas [14]. A longer response time may affect at least two (pH, lactate value) of the five rCAST score components [30,31]. The greater proportion of non-shockable rhythm in our patients makes comparing groups more difficult. Considering that the rCAST score can be influenced by EMS factors [27,29] validation of the rCAST score in specific regions or populations may be necessary before it is widely applied.

The implementation of TTM is another confounding factor for the different distribution compared to the study of Nishikimi et al. [14]. Although the American Heart Association, and European Resuscitation Council guidelines have suggested TTM for post-cardiac arrest care since 2003 [32], its implantation in different regions varies widely from 2.3%~53.2% [1]. Barriers to implementation include difficulty in interdisciplinary collaboration, lack of equipment, awareness, and agreement with guidelines, and higher priority for other aspects of care [33,34]. Nishikimi et al. used data from the Japanese Association for Acute Medicine to validate the rCAST score [14] and only 6.3% of the OHCA patients who arrived at the emergent department received TTM, possibly resulting in selection bias. Our results confirmed that the rCAST score had moderate predictive accuracy for poor neurologic outcomes and mortality; however, its efficacy may need further validation in patient groups with a higher TTM implantation rate. The results may be affected by differences in EMS practices and selection bias. 

To the best of our knowledge, this is the first validation study outside Japan using the rCAST score to predict poor neurological outcomes and mortality of OHCA patients undergoing TTM therapy. However, this study also has several limitations. First, it was conducted at a single center, which may only represent part of Taiwan’s picture. Second, the retrospective observational study design may have resulted in selection bias, including thirty-three patients without lactate value within 15 min after ROSC were excluded from analysis and deciding which PCAS patients should undergo TTM. Third, only a small number of patients were classified as high severity category of rCAST in our cohort. A larger scale calibration might be needed to confirm our findings. Fourth, the outcomes of patients were poorer in our cohort than in the Japanese cohort. Multiple factors affected the outcome of PCAS patients, including post-resuscitation care other than TTM and the composition of the patient population. It may also be due to nine patients dying after withdrawing from life support. However, the withdrawal of life support in our institute was only performed in patients with refractory organ failure and judged by two experienced physicians after discussion with the patient family surrogates. The outcomes of TTM varied largely across the different populations and studies [14,35,36]. It would be very difficult to compare the results between the different studies. Our study aimed to evaluate the predictive value of rCAST to generalize in the clinical situation other than the original population, the relatively poor outcome did not detract from the value of our study. Fifth, all the TTM performed in our hospital used surface cooling devices. Some researchers reported that endovascular cooling devices were more beneficial to the outcome of PCAS patients [37,38]. Further validation study may be considered in the patients treated with endovascular cooling devices. Finally, using cold Ringer’s lactate solution during induction of TTM may have influenced the outcomes. Cold saline infusion during resuscitation has been associated with worse outcomes [39]. However, the timing of its application in our patients was after ROSC, in line with a previous study; [5] which has been reported to be safe [40,41,42]. Despite these limitations, this study still provides further data on regional differences in the distribution of rCAST scores. 

## 5. Conclusions

In this study, the rCAST score showed moderate accuracy in PCAS patients receiving TTM to predict poor neurologic outcomes and mortality at day 28. However, the distribution of patients in each severity category according to the rCAST score was very different from the original report in Japan. A further large-scale study may be needed to calibrate the cut point of rCAST severity categories.

## Figures and Tables

**Table 1:** Basic characteristics and comparison between different hospital outcomes.

	All Patients (*n* = 108)	Good Neurologic Outcome (*n* = 16)	Poor Neurologic Outcome (*n* = 92)	*p* Value	Survival (*n* = 49)	Mortality (*n* = 59)	*p* Value
Age, years	66.0 (55.5–77.5)	54.0 (49.3–63.4)	66.0 (64.1–70.0)	0.0039	64.0 (52.8–75.0)	67.0 (59.5–80.0)	0.1133
Sex, male/female	66/42 (61.1%/38.9%)	11/5 (68.7%/31.2%)	55/37 (59.8%/40.2%)	0.4991	29/20 (59.2%/40.8%)	37/22 (62.7%/37.3%)	0.7094
Witnessed	104 (96.3%)	15 (93.7%)	89 (96.7%)	0.4786	46 (93.9%)	58 (98.3%)	0.3273
Bystander chest compression	47 (43.5%)	8 (50.0%)	39 (42.4%)	0.5728	20 (40.8%)	27 (45.8%)	0.6074
Bystander defibrillation	14 (13.0%)	6 (37.5%)	8 (8.7%)	0.0016	10 (20.4%)	4 (6.8%)	0.0457
Initial rhythm, shockable	30 (27.8%)	12 (75.0%)	18 (19.6%)	<0.0001	21 (42.9%)	9 (15.3%)	0.0015
Duration of resuscitation effort, minutes	28.0 (15.0–40.5)	14.0 (4.5–30.5)	30.0 (16.0–43.0)	0.0027	25.0 (11.0–33.0)	33.5 (17.0–46.0)	0.0033
Motor GCS score < 2	50 (46.3%)	0 (0.0%)	50 (54.3%)	<0.0001	9 (18.4%)	41 (69.5%)	<0.0001
Serum pH	7.36 (7.26–7.43)	7.35 (7.27–7.41)	7.36 (7.26–7.43)	0.9173	7.37 (7.29–7.44)	7.33 (7.19–7.41)	0.0144
Serum lactate, mg/dL	56.2 (38.8–86.7)	46.0 (37.5–85.6)	56.6 (39.9–87.3)	0.4262	53.5 (37.9–79.4)	63.0 (40.9–98.6)	0.1479
rCAST score	6.0 (2.5–8.5)	2.5 (1.5–3.8)	7.0 (3.0–9.5)	0.0001	3.0 (2.0–5.5)	8.0 (5.6–11.0)	<0.0001
Co-morbility							
Heart failure	24 (22.2%)	5 (31.2%)	19 (20.7%)	0.3489	10 (20.4%/0)	14 (23.7%)	0.6808
Old stroke	12 (11.1%)	2 (12.5%)	10 (10.9%)	>0.9999	8 (16.3%)	4 (6.8%)	0.1351
Diabetes	38 (35.2%)	3 (18.8%)	35 (38.0%)	0.1652	11 (22.4%)	27 (45.8%)	0.0119
CAD	29 (26.9%)	5 (31.2%)	24 (26.1%)	0.6686	12 (24.5%)	17 (28.8%)	0.6154
COPD/Asthma	15 (13.9%)	2 (12.5%)	13 (14.1%)	>0.9999	6 (12.2%)	9 (15.3%)	0.6541
Malignancy	8 (7.4%)	0 (0.0%)	8 (8.7%)	0.1097	4 (8.2%)	4 (6.8%)	>0.9999
ESRD on hemodialysis	12 (11.1%)	0 (0.0%)	12 (13.0%)	0.2068	4 (8.2%)	8 (13.6%)	0.5408
Cirrhosis	2 (1.9%)	0 (0.0%)	2 (2.2%)	>0.9999	0 (0.0%)	2 (3.4%)	0.4997
PCI	19 (17.6%)	5 (31.2%)	14 (15.2%)	0.1218	13 (26.5%)	6 (10.2%)	0.0269
IABP	8 (7.4%)	4 (15.0%)	1 (4.3%)	0.0161	6 (12.2%)	2 (3.4%)	0.1374
APACHEII	31.5 ± 6.3	25.3 ± 5.8	32.6 ± 5.8	<0.0001	29.8 ± 6.8	32.9 ± 5.6	0.0096

APACHE: acute physiology and chronic health evaluation, COPD: chronic obstructive pulmonary disease, CAD: coronary artery disease, ECMO: extracorporeal membrane oxygenation, ESRD: end-stage renal disease, GCS: Glasgow Coma Scale, IABP: intra-aortic balloon pump, PCI: percutaneous coronary intervention.

**Table 2:** Predictive probability of poor neurologic outcomes or mortality at day 28 according to the rCAST severity category.

(*n* = 108)	Number of Patients	Probability of Poor Neurologic Outcome	Probability of Hospital Mortality
Low severity category	53	69.8% (55.7%–81.7%)	28.3% (16.8%–42.4%)
Moderate severity category	54	100.0% (93.4%–100.0%)	79.6% (66.5%–89.4%)
High severity category	1	100.0% (2.5%–100.0%)	100.0% (2.5%–100.0%)

Data are presented as the probability (95% confidence interval).

## Data Availability

Not applicable.

## Supplementary Materials

The following supporting information can be downloaded at: https://www.mdpi.com/article/10.3390/jcm12010242/s1, Table S1: The definition of neurologic outcome according to Glasgow–Pittsburgh cerebral performance category; Table S2: Sensitivity and specificity of using the rCAST score to predict poor neurological outcomes at day 28 in out-of-hospital cardiac arrest patients; Table S3: Sensitivity and specificity of using the rCAST score to predict mortality at day 28 in out-of-hospital cardiac arrest patients.
