# Supplementary material for: Does One Size Fit All? External Validation of the rCAST Score to Predict the Hospital Outcomes of Post-Cardiac Arrest Patients Receiving Targeted Temperature Management
Source: J Clin Med. 2022 Dec 28;12(1):242. doi: 10.3390/jcm12010242 (PMC9821639; doi:10.3390/jcm12010242)
Supplement: Supplementary file 1 [file jcm-12-00242-s001.zip › jcm-2096374-supplementary.pdf]

Table S1. The definition of neurologic outcome according to Glasgow–Pittsburgh cerebral performance category. [24, 25]

CPC 1	Good cerebral performance, or only had minor psychological or neurological deficits.	Good neurologic outcome
CPC 2	Moderate cerebral disability with independent activities of daily life.	
CPC 3	Severe cerebral disability, and dependent on others for daily life.	Poor neurologic outcome
CPC 4	Coma and vegetative state.	
CPC 5	Death or brain death	

CPC: cerebral performance category

Table S2. Sensitivity and specificity of using the rCAST score to predict poor neurological outcomes at day 28 in out-of-hospital cardiac arrest patients.

rCAST score	Sensitivity	95% CI	Specificity	95% CI
≥0	100	96.1 - 100.0	0	0.0 - 20.6
>0	98.91	94.1 - 100.0	6.25	0.2 - 30.2
>0.5	95.65	89.2 - 98.8	18.75	4.0 - 45.6
>1	91.3	83.6 - 96.2	25	7.3 - 52.4
>1.5	85.87	77.0 - 92.3	25	7.3 - 52.4
>2	83.7	74.5 - 90.6	43.75	19.8 - 70.1
>2.5	79.35	69.6 - 87.1	62.5	35.4 - 84.8
>3	71.74	61.4 - 80.6	62.5	35.4 - 84.8
>3.5	68.48	58.0 - 77.8	75	47.6 - 92.7
>4	65.22	54.6 - 74.9	87.5	61.7 - 98.4
>5	64.13	53.5 - 73.9	100	79.4 - 100.0
>5.5	59.78	49.0 - 69.9	100	79.4 - 100.0
>6	54.35	43.6 - 64.8	100	79.4 - 100.0
>6.5	52.17	41.5 - 62.7	100	79.4 - 100.0
>7	48.91	38.3 - 59.6	100	79.4 - 100.0
>7.5	42.39	32.1 - 53.1	100	79.4 - 100.0
>8	30.43	21.3 - 40.9	100	79.4 - 100.0
>8.5	27.17	18.4 - 37.4	100	79.4 - 100.0
>9	26.09	17.5 - 36.3	100	79.4 - 100.0
>9.5	23.91	15.6 - 33.9	100	79.4 - 100.0
>10	20.65	12.9 - 30.4	100	79.4 - 100.0
>10.5	18.48	11.1 - 27.9	100	79.4 - 100.0
>11	11.96	6.1 - 20.4	100	79.4 - 100.0
>11.5	9.78	4.6 - 17.8	100	79.4 - 100.0
>12	7.61	3.1 - 15.1	100	79.4 - 100.0
>12.5	6.52	2.4 - 13.7	100	79.4 - 100.0
>13	4.35	1.2 - 10.8	100	79.4 - 100.0
>13.5	3.26	0.7 - 9.2	100	79.4 - 100.0
>14	1.09	0.03 - 5.9	100	79.4 - 100.0
>16	0	0.0 - 3.9	100	79.4 - 100.0

Table S3. Sensitivity and specificity of using the rCAST score to predict mortality at day 28 in out-of-hospital cardiac arrest patients.

rCAST score	Sensitivity	95% CI	Specificity	95% CI
≥0	100	93.9 - 100.0	0	0.0 - 7.3
>0	98.31	90.9 - 100.0	2.04	0.05 - 10.9
>0.5	98.31	90.9 - 100.0	12.24	4.6 - 24.8
>1	96.61	88.3 - 99.6	20.41	10.2 - 34.3
>1.5	89.83	79.2 - 96.2	22.45	11.8 - 36.6
>2	88.14	77.1 - 95.1	30.61	18.3 - 45.4
>2.5	86.44	75.0 - 94.0	42.86	28.8 - 57.8
>3	81.36	69.1 - 90.3	51.02	36.3 - 65.6
>3.5	79.66	67.2 - 89.0	59.18	44.2 - 73.0
>4	77.97	65.3 - 87.7	67.35	52.5 - 80.1
>5	77.97	65.3 - 87.7	73.47	58.9 - 85.1
>5.5	74.58	61.6 - 85.0	77.55	63.4 - 88.2
>6	71.19	57.9 - 82.2	83.67	70.3 - 92.7
>6.5	69.49	56.1 - 80.8	85.71	72.8 - 94.1
>7	64.41	50.9 - 76.4	85.71	72.8 - 94.1
>7.5	54.24	40.8 - 67.3	85.71	72.8 - 94.1
>8	42.37	29.6 - 55.9	93.88	83.1 - 98.7
>8.5	37.29	25.0 - 50.9	93.88	83.1 - 98.7
>9	37.29	25.0 - 50.9	95.92	86.0 - 99.5
>9.5	35.59	23.6 - 49.1	97.96	89.1 - 99.9
>10	30.51	19.2 - 43.9	97.96	89.1 - 99.9
>10.5	27.12	16.4 - 40.3	97.96	89.1 - 99.9
>11	18.64	9.7 - 30.9	100	92.7 - 100.0
>11.5	15.25	7.2 - 27.0	100	92.7 - 100.0
>12	11.86	4.9 - 22.9	100	92.7 - 100.0
>12.5	10.17	3.8 - 20.8	100	92.7 - 100.0
>13	6.78	1.9 - 16.5	100	92.7 - 100.0
>13.5	5.08	1.1 - 14.1	100	92.7 - 100.0
>14	1.69	0.04 - 9.1	100	92.7 - 100.0
>16	0	0.0 - 6.1	100	92.7 - 100.0
